# Use of Population-Based Health Informatics Research to Improve Care for Patients with Cardiovascular Diseases

**DOI:** 10.3390/diseases8040047

**Published:** 2020-12-17

**Authors:** Ify R. Mordi, Magalie Guignard-Duff, Christopher Hall, Benjamin Jaa Ming New, Chim C. Lang

**Affiliations:** 1 Division of Molecular & Clinical Medicine, School of Medicine, Ninewells Hospital & Medical School, University of Dundee, Dundee DD1 9SY, UK; i.mordi@dundee.ac.uk (I.R.M.); b.new@dundee.ac.uk (B.J.M.N.); 2 Health Informatics Centre, Ninewells Hospital & Medical School, Dundee DD1 9SY, UK; m.guignardduff@dundee.ac.uk (M.G.-D.); c.hall@dundee.ac.uk (C.H.)

**Keywords:** population electronic health records, renin–angiotensin system blockers, aortic stenosis, metformin, heart failure

## Abstract

There are common clinical scenarios in chronic heart disease where no randomized controlled data exist to guide management, and it is likely that well-designed observational studies will have to be used to inform clinical practice. Showing the clinical applicability of this type of study design, using record linkage of population electronic health records, we have provided key observational evidence that use of renin–angiotensin-system (RAS) blockers is associated with better outcomes in patients with aortic stenosis and that metformin could be used safely as an antiglycemic drug in patients with diabetes and heart failure. Each of these pieces of underpinning research has made a major contribution to relevant international clinical practice guidelines, helped the Food and Drug Administration in their decision making and changed prescribing practice.

## 1. Background

The digitization of health records has created an opportunity in the secondary use of routinely collected electronic health records (EHRs) for public health research in large-scale studies.

There are several common and important clinical scenarios in chronic heart disease, and it is difficult to outline an evidence-based treatment strategy because there have been no randomized trials that have adequately explored the risks and benefits of these drug therapies. Furthermore, due to the current research landscape, it is unlikely that large, adequately powered, randomized controlled trials (RCTs) will be undertaken in the future. In these populations, no RCT-level data exist to guide management, and clinicians can only be guided by well-designed observational studies.

Demonstrating one potential approach to this problem, we have linked our EHRs with cardiac investigations including echocardiograms and utilized these enriched EHRs to provide key population cohort data to demonstrate the safe use of drugs in conditions in which their use had previously been avoided due to what were presumed to be classical contraindications for use, described in the British National Formulary and the manufacturers’ prescribing information (Physicians’ Desk Reference) in two case scenarios: “Use of renin angiotensin system (RAS) blockers are contraindicated for use in patients with aortic stenosis” (AS); and “metformin should be used with caution in patients with type 2 diabetes mellitus (T2DM) and co-existing heart failure” (HF). Because of these contraindications, these drugs have been little studied in RCTs. Despite the prevailing clinical treatment paradigms at the time, there was no definitive trial evidence to support these contraindications, and importantly, there were plausible reasons to believe that patients might actually derive benefits from these treatments.

## 2. Scenario 1: RAS Inhibition in Aortic Stenosis

AS is the most common form of valvular heart disease in the western world and affects 2% to 4% of adults older than age 65 years. It is typically a progressive disease with a long asymptomatic phase, but once symptoms develop, the prognosis is poor, with 50% mortality at one year [1]. In patients with asymptomatic AS, hypertension is a common comorbidity found in up to 78% of patients, associated with a 56% higher rate of cardiovascular events and two-fold increased mortality [2]. There was uncertainty about what drugs should be used to treat concomitant hypertension in AS, especially concerning the use of RAS blockers that were perceived as being relatively contraindicated due to their vasodilatory effect. However, it is increasingly recognized that AS incorporates a disease process that extends beyond the valve itself, leading to an aortic valvular ‘heart’ disease with adverse left ventricular modeling, which may be related to the RAS. We previously reported the findings of population-based data that investigated the impact of RAS blockers on outcomes in AS [3]. In brief, the Health Informatics Centre dispensed prescribing, morbidity and mortality database for the population of Tayside, Scotland was linked through a unique patient identifier to the Tayside echocardiography database. This population-based research identified 2117 patients with AS, with a mean follow-up of 4.2 years. This cohort featured 1585 (75%) patients with nonsevere and 532 (25%) with severe AS. A total of 699 patients were on RAS blockers. The study showed that the use of RAS blockers was associated with a significant reduction in cardiovascular events (HR: 0.77, 95% confidence interval: 0.65 to 0.92, *p* < 0.0001) and all-cause mortality (HR: 0.76, confidence interval: 0.62 to 0.92, *p* < 0.0001) [3]. This Tayside study was the first to provide key observational evidence that ACEI or ARBs are not only safe to use but may be associated with improved outcomes. These protective effects of RAS in AS have since been confirmed by other groups [4,5].

## 3. Scenario 2: Patients with Type 2 Diabetes and Heart Failure: Use of Metformin and Importance of Glycemic Control

In HF cohorts, the prevalence ranges from 10 to 47%, and in patients with T2DM, the prevalence ranges from 9 to 22% [6]. T2DM is independently associated with a greater risk of death and rehospitalization. Treating patients with concomitant HF and T2DM can be challenging. It has been difficult to outline an evidence-based diabetic treatment strategy as there have been no randomized trials adequately exploring the risks and benefits of diabetic therapies in this population. Additionally, controversy exists regarding the importance of glycemic control in patients with T2DM and HF based on conflicting reports using single-baseline glycosylated hemoglobin (HbA1c). Metformin was previously contraindicated in patients with HF because of concerns of lactic acidosis, although this may reflect previous experience with phenformin-associated lactic acidosis. With respect to this, we published a retrospective population cohort study utilizing record linkage of EHRs of the incidence of contraindications to the use of metformin and the proportion that discontinued metformin treatment following the development of a contraindication in patients with T2DM from January 1993 to June 1995 [7]. The study showed that 24.5% of patients prescribed metformin had contraindications to its use. The development of contraindications rarely resulted in the discontinuation of metformin therapy, and lactic acidosis was rare (one episode of lactic acidosis in 4600 patient years). In another study examining the use of metformin in T2DM and HF, we found that among 427 T2DM patients with incident HF, fewer deaths occurred in metformin users, alone or in combination with the sulfonylurea monotherapy cohort at one year (HR of 0.59 (0.36–0.96)) and over long-term follow-up (HR, 0.67 (0.51–0.88)) [8].

To explore the importance of glycemic control, we previously published a study reporting the relationship between time-weighted mean HbA1c and all-cause death after HF diagnosis in a population cohort of 1447 patients with HF diagnosed after T2DM that also had HbA1c measurements. The Cox regression model with an adjusted hazards ratio reported a U-shaped curve between HbA1c and mortality in patients with T2DM and HF, with the lowest risk in patients with moderate glycemic control (HbA1c 7.1–8.0% HR 1.0) [9].

In this report, we explored the impact of our published key observational evidence on these two scenarios. Specifically, we explored their potential contribution to changes in prescribing practice and in relation to the publication of the research findings and changes in clinical practice guidelines.

## 4. Methods

This was a retrospective, population-based, longitudinal study conducted in the population of Tayside, Scotland (approximately 400,000) using the dispensed prescribing database maintained by the Health Informatics Centre (HIC), University of Dundee and NHS Tayside. This contains records of all dispensed community prescriptions dating back to 1993. Clinical information was collected according to the national clinical dataset for the care of patients in Scotland. Datasets available from HIC also included hospital discharge data, biochemistry data, mortality data, sociodemographic descriptors and other data that are linked by a unique 10-digit patient identifier, the community health index number, that is used for all healthcare activities in Tayside. Access to the anonymized and validated clinical datasets was administered by HIC using established protocols approved by the Research Ethics Committee in Tayside.

In Case Scenario 1, all patients with a diagnosis of moderate to severe AS between January 2000 and December 2019 were identified from the Tayside echocardiogram database. The diagnosis and severity of AS were ascertained by the British Society of Echocardiography accredited echocardiographers at the time of the scan. We then identified those who were newly prescribed a RAS blocker in this cohort during the study period. The prescribing of RAS blockers constituted at least 2 prescriptions of an angiotensin-converting enzyme inhibitor or an angiotensin receptor blocker.

In Case Scenario 2, we identified all Tayside residents with a diagnosis of both T2DM and HF between January 2000 and December 2019. We then identified all those who were newly treated with metformin during the study period. The prescribing of metformin constituted at least 2 prescriptions of metformin.

### 4.1. Relationship to Clinical Practice Guidelines and Drug Regulations

To help identify any changes in prescribing practice, we will display prescribing data in relation to the timing of dissemination of the research findings in scientific presentations at meetings, publications and when the research was included in clinical practice guidelines.

### 4.2. Impact on Clinical Practice Guidelines

Our research has impacted international clinical practice guidelines that are used by healthcare decision-makers to optimize and improve care. Our research provided the key underpinning evidence used in their recommendations for use in patients with aortic stenosis. Hypertension is common and harmful in patients with AS. Medical therapy is now strongly recommended (Class I; Level B). Our research [3] was cited as the underpinning evidence for the use of RAS blockers as ‘an antihypertensive agent in patients with AS’ (Class 1, Level of Evidence: B) in the 2014 ACC/AHA guidelines [10].

Our research has also impacted clinical practice guidelines for T2DM and HF. Our EHRs research demonstrated that metformin was not only safe to use [6] but may be associated with improved survival in patients with T2DM and HF [7]. Our research provided the evidence that guided the recommendation by international CPGs regarding the safe use of metformin in achieving glycemic control in these patients, including the 2011 update to the National Heart Foundation of Australia and Cardiac Society of Australia and New Zealand Guidelines for the prevention, detection and management of chronic heart failure [11] and 2016 American Heart Association Scientific Consensus Statement and 2017 ACC/AHA/HFSA Statement [12,13]. We have provided the underpinning evidence for practice advice on glycemic control in this vulnerable patient group, and the guidelines recommend ‘a target range of HbA1c 7% to 8% for most patients with HF’ [13,14,15].

### 4.3. Impact on Regulatory Guidance

We also explored if our research impacted decision making by regulatory authorities. In this respect, our research demonstrating the safety of metformin was instrumental in the lifting of warnings on the use of metformin by the FDA in 2016 [16].

## 5. Results

**Figure 1:**
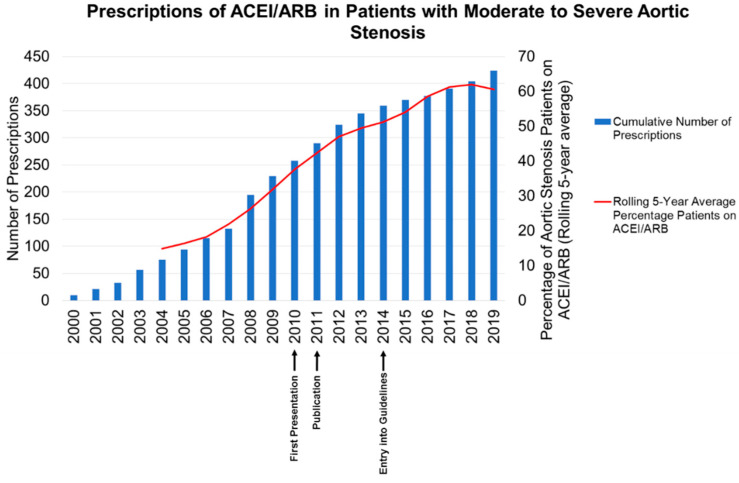
Prescribing of ACEI/ARB in patients with moderate to severe aortic stenosis in Tayside between 2000 and 2019.

Case Scenario 1. We found an increase in the number of prescriptions of RAS blockers in patients with moderate to severe aortic stenosis, with an increase in the percentage of patients with moderate to severe AS on RAS blockers (Figure 1). Figure 1 also indicates the timing of local/international presentations [17,18], publication of the results and mention in clinical practice guidelines.

**Figure 2:**
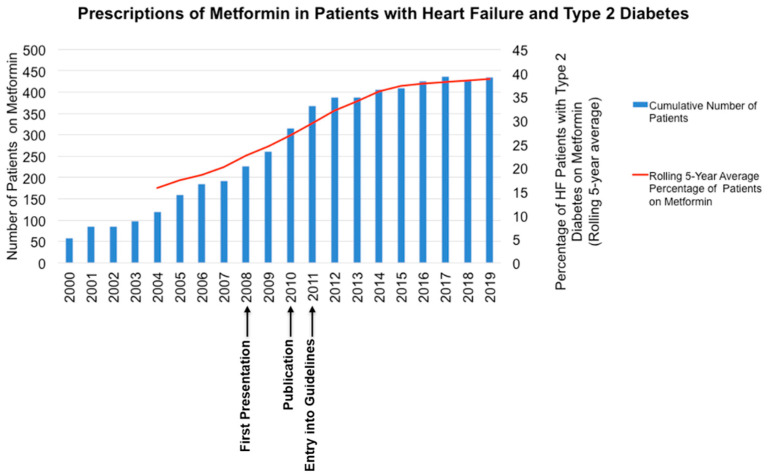
Prescribing of metformin in patients with heart failure and type 2 diabetes in Tayside between 2000 and 2019.

Case Scenario 2. We found an increase in the number of prescriptions of metformin in patients with T2DM and HF, with an increase in the percentage of patients with moderate to severe AS on metformin (Figure 2). Figure 2 also indicates the timing of local presentation [19], international presentation [20], publication of the results and entry in clinical practice guidelines.

## 6. Discussion

In this report, we have shown an increase in prescribing both RAS blockers in patients identified to have moderate to severe AS and metformin in patients with HF and T2DM. There has been a gradual increase in the use of both drug classes in the two case scenarios over the years. The observational nature of the study means that we cannot confidently attribute this change in prescribing practice to our research findings. Although it is likely that our research might have likely helped to increase its wider use in the two case scenarios, we cannot exclude that other events may have occurred that could influence the increase in prescribing of these drugs.

In conclusion, we have shown the potential of population-based EHRs to impact and provide a major contribution to healthcare, as evident from the impact on relevant clinical practice guidelines internationally, which helped the Food and Drug Administration in their decision making and changed prescribing practice, demonstrating the power of this type of research to influence clinical practice in areas where randomized clinical trial data are unlikely to be forthcoming. It should also be noted that with the advances in EHRs and the proliferation of genomic research in health systems, there is potential for population-based EHRs to provide insights into emerging paths for the widespread implementation of personalized medicine [21].

## Disclosures

IMSupported by an NHS Education for Scotland/Chief Scientist Office Postdoctoral Clinical Lectureship (PCL 17/07)M G-DNoneCHNoneBJMNNoneCCLDeclares receiving consultancy fees and/or research grants from Amgen, AstraZeneca, MSD, Novartis, and Servier; grant funding from European Union Horizon 2020 IMI, MRC, British Heart Foundation, EFSD.
